# Short-term increases in rival number improves single mating productivity in male *Drosophila*

**DOI:** 10.1093/beheco/araf032

**Published:** 2025-04-10

**Authors:** Lily Amos, Stuart Wigby, Liam R Dougherty

**Affiliations:** Department of Evolution, Ecology and Behaviour, University of Liverpool, Crown Street, Liverpool, L69 7RB, UK; Department of Evolution, Ecology and Behaviour, University of Liverpool, Crown Street, Liverpool, L69 7RB, UK; Department of Evolution, Ecology and Behaviour, University of Liverpool, Crown Street, Liverpool, L69 7RB, UK

**Keywords:** behavioural plasticity, cryptic female choice, Drosophila melanogaster, multiple stressors, reproductive plasticity, sperm competition

## Abstract

In variable environments, animals can change their reproductive behaviors and physiology to maximize reproductive returns. Natural environments vary in multifaceted ways, and animals may need to integrate multiple social or physical cues to adopt the most effective behavioral strategy. In a fully factorial 2 × 2 × 2 experiment, we exposed males to three factors: the number of rivals (10 or 30), food availability (present/absent) and mechanical shaking (present/absent). After 60 min of exposure, we recorded the male’s mating latency, copulation duration and the number of offspring produced after a single mating. We also noted the latency of the males partner to remate with a stock male 24 h later. When rival number was increased from 10 per vial to 30 per vial, males sired more offspring. Males also varied their copulation duration and mating latency in response to the number of rivals, but in a condition-dependent manner. In the absence of vortexing, males mated for a shorter time when kept with 30 rivals, but the opposite was observed when males were vortexed. When males were fed and held in groups of 30, they took longer to begin mating compared to the other treatments. Our findings are consistent with the idea that male *Drosophila* integrate social cues to respond to levels of sperm competition and plastically allocate their ejaculate, but we have demonstrated that they can occur more rapidly (1 h) than previously thought (>24 h). Overall, our data highlight that combinatorial approaches can reveal new relationships between environment and behavior.

## Teaser

Animals adjust their reproductive strategies based on their environment to maximize their fitness. In the fruit fly, males produce more offspring when exposed to increased numbers of rival males before mating. Being in physical danger and the availability of food can change how males adjust their mating duration and mating latency in response to the number of rivals. Surprisingly, these changes can happen within an hour, which is faster than we previously thought.

## Introduction

Since the natural environment is highly variable, animals must respond to social and physical cues and express different phenotypes depending on current conditions; a process known as phenotypic plasticity (West-Eberhard 2003). Reproductive behavior and physiology are thought to be especially plastic in response to changes in the environment, because reproductive decisions can have large fitness consequences. One common way in which males exhibit reproductive plasticity is through the size of their ejaculates. Strategic ejaculation makes the best use of limited sperm and seminal fluid that can become depleted through repeated matings (Wedell et al. 2002; Sirot et al. 2009; Reinhardt et al. 2011). Sperm and seminal fluid depletion is associated with reduced fertilization success (Fuyuo 1981; Preston et al. 2001; Linklater et al. 2007), and the ejaculate can take significant time and energy to replenish (Sirot et al. 2009; Vahed et al. 2011; Friesen et al. 2015). Hence, males often experience a trade-off between investment into current versus future matings (Parker 1982). Sexual selection therefore favors males who allocate their ejaculates prudently and strategically (Parker and Pizzari 2010).

A stressor is any environmental factor that has the potential to compromise an individual’s fitness (Schulte 2014), and natural environments contain many potential stressors. Accordingly, animals should demonstrate plasticity to minimize fitness loss in instances of stress. One well-studied environmental cue is the perceived level of intraspecific competition. The number of same-sex individuals may signal the number of rivals competing for matings (Bretman et al. 2011) or (when females mate multiply) for fertilisations (Parker 1970; Simmons 2002). Theory suggests that males should increase their investment in reproduction when competition is present, but conserve their resources when competition is absent (Parker 1993; Parker et al. 1997). Across taxa, males have been shown to strategically alter their courtship behavior (Bertram et al. 2013), the quality and quantity of their ejaculate (Kelly and Jennions 2011; Fitzpatrick and Lüpold 2014), and their copulation duration (Bretman et al. 2009; Nandy and Prasad 2011) in response to the number of rivals.

Another important environmental cue is the level of food or water. Short periods without food may cause individuals to prioritize food-seeking behaviors at the expense of reproduction (Limbania et al. 2023). Long periods without food or water leads to starvation, desiccation, and ultimately death. To compensate for this, individuals in a starved or desiccated state may demonstrate terminal investment, where they invest heavily in their current matings to maximize their fitness (Clutton-Brock 1984; Duffield et al. 2017). Therefore, there is evidence that males may respond to a reduction in food or water availability by either increasing or decreasing their investment in reproduction, depending on the severity of the stress.

Another well-established form of stress is mechanical stress. Although somewhat artificial, vortexing/shaking has been used in laboratory experiments to induce an array of physiological stress responses in insects (Suh et al. 2004; Neckameyer and Weinstein 2005; Chen et al. 2008; Mowlds et al. 2008; Yost et al. 2021). For example, when the fruit fly, *Drosophila melanogaster*, is vortexed, individuals release a stress odor for up to 1 h, which other flies actively avoid (Suh et al. 2004; Yost et al. 2021). Exposure to vortexing can also affect dopamine production, locomotion (Neckameyer and Weinstein 2005), learning and memory (Chen et al. 2008). Some of these responses have been shown to require neural activity (Suh et al. 2004) which demonstrates they are active changes in response to an environmental stimulus. However, there is very little literature exploring the direct effects of vortexing on mating behaviors and physiology in males. Mechanical stress may mimic physical danger; if animals perceive that they are in physical danger, it is likely that mate-seeking behaviors would increase as a form of terminal investment (Clutton-Brock 1984; Duffield et al. 2017).

The effects of single stressors on male reproductive decisions have been well explored (see above). However, wild animals live in complex environments, and fluctuations in one stressor are likely to occur simultaneously or sequentially with others. Despite this, a review by Kaunisto et al. (2016) found fewer than ten experimental studies that manipulated three stressors in a fully factorial design. Further, individual stressors can interact in several ways (eg additive, synergistic and antagonistic), so the effects of single stressor studies cannot simply be combined to predict their outcomes. Furthermore, it has also been shown that reproductive plasticity can occur more rapidly when the “intensity” of a cue is stronger (Carazo et al. 2012; Lymbery et al. 2019). Therefore, when multiple stressors are experienced simultaneously, behavioral changes may be seen faster than previously shown for single stressors. Since the diversity and intensity of stressors in nature are increasing due to human activity and the resulting climate change, estimating how multiple stressors may interact is of particular importance.

Here we tested the effect of three factors on male reproductive strategies in the fruit fly, *Drosophila melanogaster*. First, we manipulated the level of mechanical shaking via vortexing, in order to induce a stress response. Vortexing *D. melanogaster* causes the release of an alarm pheromone called the *Drosophila* stress odor (Suh et al. 2004), and reduces locomotor activity in males for at least 15 min (Neckameyer and Weinstein 2005). Second, we manipulated the males’ access to food prior to mating, because *D. melanogaster* have been shown to initiate rapid physiological and behavioral responses to the absence of food (Arya et al. 2021; Cheriyamkunnel et al. 2021; Yost et al. 2021). The presence of food is likely to be an important environmental cue for *D. melanogaster* because laboratory populations get both their nutrients and water from the fly food, as well as being the substrate in which their offspring develop. Third, we manipulated the males’ social environment, because in response to an elevated risk of competition, *D. melanogaster* males can adaptively increase their sperm quality, quantity and ejaculate size (Garbaczewska et al. 2013; Moatt et al. 2014; Hopkins et al. 2019b), change their ejaculate composition (Wigby et al. 2009; Fedorka et al. 2011; Hopkins et al. 2019b), and mate for longer (Bretman et al. 2009; Churchill et al. 2020). Although these environmental cues and their responses have been explored in *D. melanogaster*, how they interact to affect reproductive decisions has not. We measured the copulation duration and mating latency of males, the remating latency of their mates, and the number of offspring produced by their mates, to assay male reproductive decisions. Copulation duration is largely under male control in *D. melanogaster* (Gromko et al. 1991; Bretman et al. 2013a) and longer matings often result in increased paternity (Bretman et al. 2009, 2012; Wigby et al. 2009; Price et al. 2012; Garbaczewska et al. 2013; Dobler and Reinhardt 2016). Mating latency depends on several factors in *D. melanogaster*, including female receptivity (Manning 1967), male courtship vigour (Talyn and Dowse 2004), and male attractiveness (Poissonnier et al. 2024). The remating latency can also be directly influenced by males, because the transfer of seminal fluid proteins (SFPs) during mating can decrease the likelihood of remating (Chapman et al. 2003; Liu and Kubli 2003; Fricke et al. 2009; Fowler et al. 2022). Similarly, the rate of offspring production is under both male and female influence in *D. melanogaster*, as the transfer of male SFPs can increase egg production and impact sperm usage (Herndon and Wolfner 1995; Heifetz et al. 2000; Chapman et al. 2003; Liu and Kubli 2003; Hopkins et al. 2019a). We predicted that: a) males that were vortexed would exhibit similar mating behavior as control males, because a 1-h recovery period after vortexing is long enough for the emission of the stress odor to subside (Yost et al. 2021), b) males that were held without food/water would be less motivated to mate (and so show a longer mating latency), instead prioritizing food and water seeking over reproduction (Cheriyamkunnel et al. 2021), and c) males that were held with more rivals would mate for longer, and their partners would have longer remating latencies and produce more offspring, because higher levels of male competition would stimulate males to increase the quality and quantity of their ejaculates. We also predicted that the response of males may depend on interactions between all three factors, although, we had no a priori predictions about the strength or direction of these interactions.

## Methods

### Fly stocks and handling

Large numbers of wildtype Dahomey *Drosophila melanogaster* flies (stock population originally collected from Benin in the 1970s (Puijk and De Jong 1972)) were allowed to lay eggs on grape juice agar plates (50 g agar, 600 ml red grape juice, 42.5 ml Nipagin (10% w/v solution), per liter of medium), supplemented with fresh yeast paste. 15 μl of PBS containing eggs was then transferred to glass bottles containing a standard sugar yeast (SY) medium (100 g brewer’s yeast, 100 g sugar, 20 g agar, 30 ml Nipagin (10% w/v solution), and 3 ml propionic acid per liter of medium), and flies allowed to develop. All flies were maintained in a controlled environment at 25 °C with a 12:12 h light: dark cycle. When the adult flies began to emerge approximately 9 d later, virgin males and females were collected within 6 h of eclosion under ice anesthesia. Individuals were housed in same-sex groups of 10 in plastic SY food vials (75 × 25 mm, each containing 8 ml of medium) supplemented with grains of yeast, to age for 5 (±2) d before experimental treatments were applied.

### Treatments

**Fig. 1.**
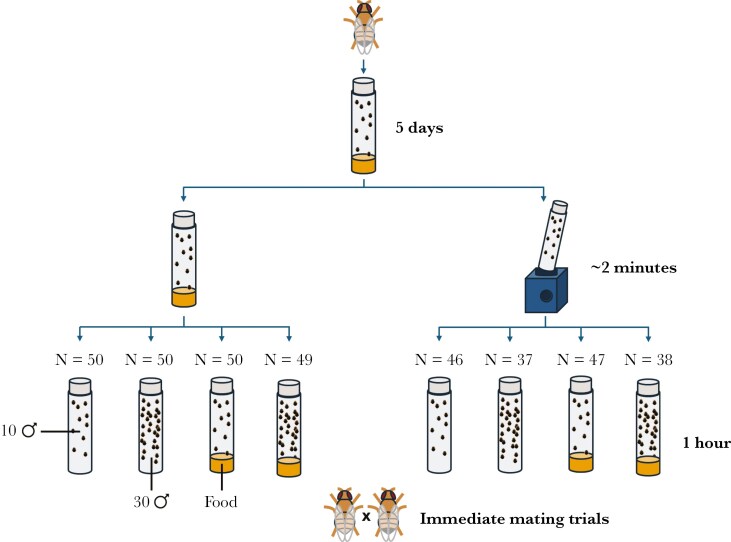
Experimental design of the male stressor treatments. Male Dahomey *Drosophila melanogaster* were either vortexed in a small falcon tube (moderate speed for 20 s, three times, with a 20 s rest between each vortex), or left in their housing vials. After vortexing, all males were left to recover for 1 h in plastic vials before doing behavioral assays. During this hour, they were either housed in groups of 10 or groups of 30 males and with or without food. Males were vortexed with the same number of rivals as they were held with for the recovery period. Sample sizes are shown above each treatment.

The morning of the mating trials, focal males (those that received environmental manipulations and were only used in mating trials) were removed from the temperature-controlled room before lights-on and randomly assigned to one of eight experimental treatments (**Fig. 1**). Treatments consisted of three factors in a fully factorial design: vortexing, food availability, and the number of rivals.

#### Vortexing

First, the morning of the mating trials, half of the focal males were vortexed and half of the focal males remained in their housing vials. Half of the males to be vortexed were transferred to 15 ml falcon tubes in the same groups of 10 they had been housed with, and half were transferred to falcon tubes in groups of 30, combining three of the housing vials. The falcon tubes did not contain any food. We did not vortex males singularly because of time constraints. Males were vortexed at a moderate speed (speed 1 of the vortex section of a Labnet VX100 vortex) for 20 s, three times, with a 20 s rest between each round. Based on observations, no flies were harmed during the vortexing process. Males were left for 1 h after vortexing to recover before behavioral analysis was carried out, to allow for the emission of the stress odor to subside (Yost et al. 2021). The recovery period began after the final males had been vortexed.

#### Food availability

Second, during the 1-h recovery period after vortexing, all focal males (including those that had not been vortexed) were transferred to new plastic vials. Half the males were transferred to vials containing SY food and half to vials without food.

#### Number of rivals

Additionally, during the 1-h recovery period when males were transferred to new plastic vials (with or without food), half of the focal males were held in groups of 10 and the other half were held in groups of 30 males. We chose these as our treatment groups because we wanted to simulate social environments males may encounter in nature (Morimoto and Pietras 2020). Males were vortexed with the same males that they were housed with during the recovery period. Treatments were blinded before the mating trials were conducted by randomly relabeling the vials. Once all focal males were in the treatments, they were returned to the temperature-controlled room before lights-on.

### Mating trials

Females were aspirated individually into fresh plastic SY vials the day prior to the mating trial. Mating trials were conducted at 25 °C, starting 30 mins after the females experienced lights-on the following day. On the morning of the mating trials, single focal males were aspirated individually into vials containing a single female. Pairs were observed in person for 2 h. We recorded the latency to mating from when the pair were introduced. We also recorded the duration of any matings. Matings began when the male mounted the back of the female and matings ended when the male dismounted the female and the genitalia disengaged. Any pairs that did not mate within 2 h were excluded from the remating trials (see below). Mated females were removed immediately after mating and placed into new individual plastic vials with live yeast grains to oviposit for 24 h. The vials were then left for 13 d to allow offspring to emerge from pupae, before freezing and counting. Two replicates of the mating trials were conducted (N_1_ = 174, N_2_ = 193, where N is the number of focal males, with one focal male per vial).

### Female remating trials

To test for differences in the remating latency of the mates of focal males, mated females were entered into remating trials 24 h after the first mating. Standard virgin males (those that did not receive environmental manipulations and were only used in remating trials) were aspirated individually into fresh glass SY vials the day prior to the remating trials. On the morning of the remating trials, females were aspirated individually into vials containing a single standard male. Pairs were observed in person for 4 h and we recorded the latency to remate from the time the pair were introduced.

### Statistical analysis

Statistical analyses were conducted in R v 4.2.3 (R Core Team 2023). Cox proportional hazards models were used to analyze mating latencies, fitted using the coxph function in the “survival” package v 3.5.3 (Therneau and Grambsch 2013; Therneau 2023). Pairs that did not mate within 120 min in the first matings, and within 240 min in the rematings, were treated as censors. One mating that was shorter than 6 min was excluded from the analysis, as sperm is unlikely to have been transferred within that time (Gilchrist and Partridge 2000). One mating that was longer than 30 min was winsorized (replaced with the next largest value rather than being removed; Wilcox 2005), using the “DescTools” package v 0.99.54 (Signorell 2025), as it is likely the mating had finished but the genitalia had failed to disengage. Three observations were excluded from the offspring production dataset because the female was mistakenly not removed after 24 h. We compared the proportion of matings which produced any offspring across the eight experimental treatments using generalized linear models with a binomial link. We compared offspring numbers (for the subset of females that produced offspring) and copulation durations across the eight experimental treatments using general linear models. We started with maximal models that included all two- and three-way interactions between vortexing, food and the number of rivals. We also included replicate as a fixed factor, rather than a random factor, because we only had two levels which is insufficient to estimate variance (Bolker et al. 2009). The time since the start of the recovery period (after vortexing, during which the males were exposed to the rival and food treatments) was included as a fixed effect, because there was variation in the start time of the mating trials. This was due to the time it took to transfer individual males from their experimental treatments to the mating vials. Then, to determine the final model, all non-significant two- and three-way interactions were dropped, provided that fits of the original and reduced models did not significantly differ following a likelihood ratio test. We present the maximal models in the supplementary materials (Tables S1-S10). Assumptions of the models were checked visually using q-q plots.

## Results

### Latency of the first mating

Using a cox survival analysis, there was no significant effect of vortexing on the interaction between food and the number of rivals affecting the latency of the first mating (X^2^ = 0.34, *p* = 0.56; electronic supplementary material, Table S1). There was, however, a marginally significant interaction between food and the number of rivals (X^2^ = 3.96, *p* = 0.05). We, therefore, removed all other non-significant interactions from our final model (all p > 0.05; electronic supplementary material, Table S2).

**Fig. 2.**
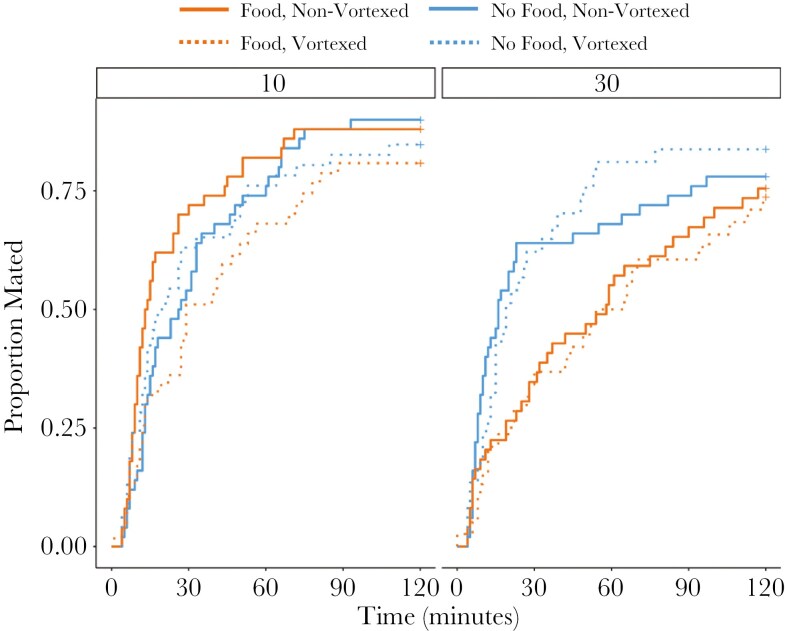
Mating latency in response to male food and rival number treatments prior to copulation. Males held with 30 rivals and provided with food, tended to be slower to begin mating than other treatments.

Males that were fed tended to take longer to begin mating than males housed without food when held in groups of 30, but not in groups of 10 (X^2^ = 3.62, *p* = 0.06, Fig. 2, Table 1), though this effect was marginally non-significant. Mating latency was not influenced by vortexing (X^2^ = 0.89, *p* = 0.35). Matings in the first and second replicate did not differ in mating latency (X^2^ = 1.42, *p* = 0.23) and the time since the start of the recovery period also had no effect (X^2^ = 0.55, *p* = 0.46, Table 1).

**Table 1.** Parameter estimates from a cox proportional hazards model predicting the latency of the first mating (how long it took pairs of flies to start mating after they had been introduced). Chi-square values and their *p*-values were calculated with likelihood ratio tests.

Predictors	HR	SE	*p*-value	X^2^	*p*(X^2^)
Vortexing	0.89	0.12	0.35	0.89	0.35
Food	1.58	0.36	0.20	1.65	0.20
No of rivals	1.43	0.36	0.33	0.97	0.33
Replicate	0.87	0.12	0.23	1.42	0.23
Recovery period	1.00	0.00	0.46	0.55	0.46
Food * No of rivals	0.64	0.23	0.06	3.62	0.06

### Copulation duration

There was no significant effect of vortexing on the interaction between food and number of rivals affecting the duration of the first mating (X^2^ = 1.18, *p* = 0.28; electronic supplementary material, Table S3). There were also no significant two-way interactions between vortexing, food and the number of rivals (all p > 0.1; electronic supplementary material, Table S4), except for a significant interaction between vortexing and the number of rivals (X^2^ = 6.67, *p* = 0.01; electronic supplementary material, Table S4). We, therefore, removed all interactions except for the vortexing by rival number interaction from our final model.

**Fig. 3.**
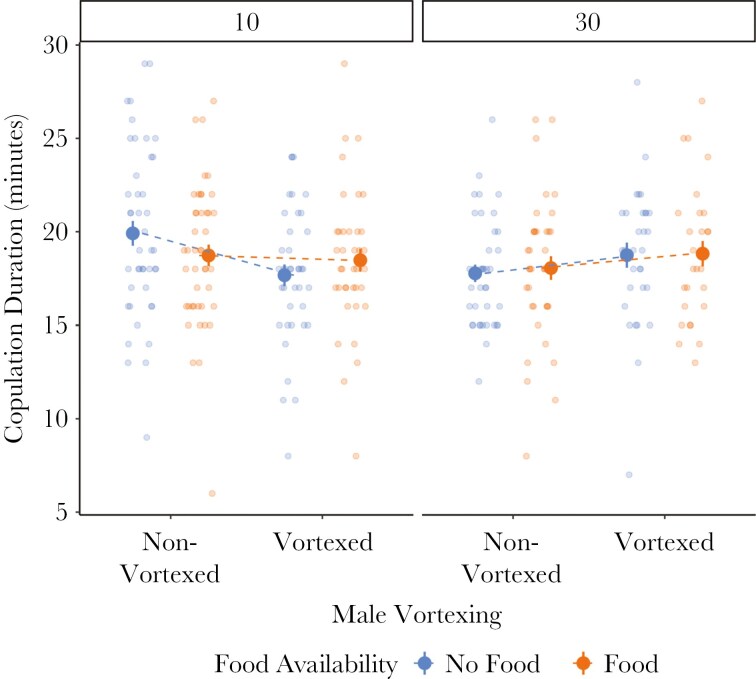
The effect of multiple stressors on mating behavior. Centre points represent means (±SE) plotted alongside raw data. The length of the first mating depended on an interaction between vortexing and the number of rivals, visually represented by dashed lines.

Vortexing caused males to mate for a shorter time after being held with 10 rivals, but mate for longer after being held in groups of 30 (X^2^ = 7.24, *p* = 0.01, Fig. 3, Table 2). Copulation duration was not affected by food availability (X^2^ = 0.12, *p* = 0.73). Matings in the first and second replicate did not differ in copulation duration (X^2^ = 3.60, *p* = 0.06) and the time since the start of the recovery period had no effect (X^2^ = 0.98, *p* = 0.32, Table 2).

**Table 2.** Parameter estimates from a linear model predicting the duration of the first mating. *F*-values and their *p*-values were calculated with likelihood ratio tests. Significant results are in bold.

Predictors	Estimate	SE	*p*	*F*	*p(F)*
Intercept	20.61	0.98			
Vortexing (Vortexed)	−1.42	0.60	**0.02**	5.62	**0.02**
Food (Food)	−0.15	0.44	0.73	0.12	0.73
No of rivals (30)	−1.55	0.60	**0.01**	6.60	**0.01**
Replicate (2)	−0.83	0.44	0.06	3.60	0.06
Recovery period	−0.01	0.01	0.32	0.98	0.32
Vortexing (Vortexed) * No of rivals (30)	2.62	0.97	**0.01**	7.24	**0.01**

### Fertility and offspring number of first mating

There was no significant effect of vortexing on the interaction between food and number of rivals affecting the proportion of first matings that produced any offspring (X^2^ = 0.0001, *p* = 0.99; electronic supplementary material, Table S5). There were also no significant two-way interactions between vortexing, food and the number of rivals (all p > 0.1; electronic supplementary material, Table S6). We, therefore, removed all interactions from our final model to report main effects.

The proportion of matings that produced any offspring did not depend on the number of rivals (X^2^ = 1.22, *p* = 0.27), vortexing (X^2^ = 1.94, *p* = 0.16), or food availability (X^2^ = 0.06, *p* = 0.80, Fig. S1, Table 3). Matings in the first and second replicate did not differ in the proportion of matings that produced any offspring (X^2^ = 0.30, *p* = 0.58) and the time since the start of the recovery period also had no effect (X^2^ = 0.87, *p* = 0.35, Table 3).

**Table 3.** Parameter estimates from a binomial model predicting the proportion of first matings that produced any offspring. *F*-values and their *p*-values were calculated with likelihood ratio tests.

Predictors	Estimate	SE	*p*	*F*	*p(F)*
Intercept	4.42	1.42			
Vortexing (Vortexed)	−0.99	0.71	0.16	1.94	0.16
Food (Food)	0.17	0.70	0.80	0.06	0.80
No of rivals (30)	0.84	0.76	0.27	1.22	0.27
Replicate (2)	0.37	0.67	0.58	0.30	0.58
Recovery period	−0.01	0.01	0.35	0.87	0.35

In the subset of females that did produce offspring, there was no significant effect of vortexing on the interaction between food and the number of rivals affecting the number of offspring females produced (X^2^ = 0.63, *p* = 0.43; electronic supplementary material, Table S7). There were also no significant two-way interactions between vortexing, food and the number of rivals (all p > 0.1; electronic supplementary material, Table S8). We, therefore, removed all interactions from our final model to report main effects.

**Fig. 4.**
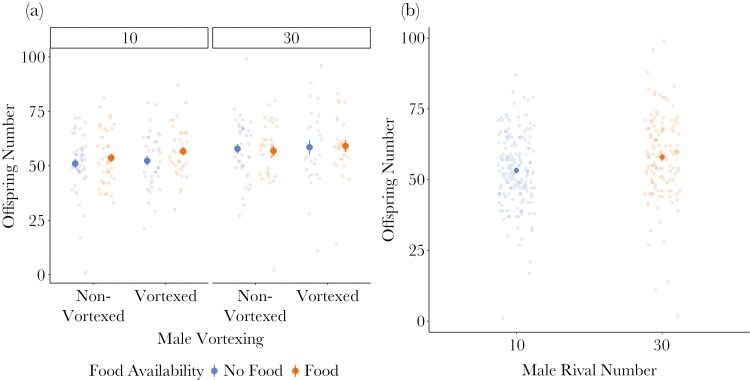
Offspring number in response to multiple stressors. Centre points represent means (±SE) plotted alongside raw data. Females that did not produce any offspring were excluded. A) Vortexing and food availability had no effect on the number of offspring produced. B) The number of offspring produced depended on the number of rivals males were housed with prior to copulation.

In the subset of females that did produce offspring, males held in groups of 30 sired significantly more offspring than males held in groups of 10 (X^2^ = 9.45, *p* = 0.002, Fig. 4, Table 4). The number of offspring was not affected by vortexing (X^2^ = 1.91, *p* = 0.17), or food availability ((X^2^ = 0.78, *p* = 0.38, Table 4). Males in the second replicate sired significantly more offspring than males in the first replicate (X^2^ = 4.38, *p* = 0.04, Table 4) and as the time since the start of the recovery period increased, the offspring number decreased (X^2^ = 3.85, *p* = 0.05, Table 4).

**Table 4.** Parameter estimates from a linear model predicting the number of offspring produced by females. This analysis was only run on the subset of females that produced offspring. *F*- values and their *p*-values were calculated with likelihood ratio tests. Significant results are in bold.

Predictors	Estimate	SE	*p*	*F*	*p*(*F*)
Intercept	54.54	3.04			
Vortexing (Vortexed)	2.28	1.65	0.17	1.91	0.17
Food (Food)	1.45	1.64	0.38	0.78	0.38
No of rivals (30)	5.05	1.64	**0.002**	9.45	**0.002**
Replicate (2)	3.40	1.63	**0.04**	4.38	**0.04**
Recovery period	−0.04	0.02	**0.05**	3.85	**0.05**

### Remating latency

Using a cox survival analysis, there was no significant effect of vortexing on the interaction between food and the number of rivals affecting the latency of the second mating (X^2^ = 1.49, *p* = 0.22; electronic supplementary material, Table S9). There were also no two-way interactions between vortexing, food and the number of rivals (all p > 0.1; electronic supplementary material, Table S10). We, therefore, removed all interactions from our final model.

Female remating latency was not affected by the number of rivals her first mate was housed with (X^2^ = 0.14, *p* = 0.71, Fig. S2, Table 5). Whether the female’s first mate was vortexed did not affect the remating latency (X^2^ = 0.01, *p* = 0.93), nor did the first male’s food availability prior to mating (X^2^ = 0.49, *p* = 0.48). Matings in the first and second replicate did not differ in the female’s remating latency (X^2^ = 0.22, *p* = 0.64) and the time since the start of the first male’s recovery period had no effect (X^2^ = 0.01, *p* = 0.91, Table 5).

**Table 5.** Parameter estimates from a cox proportional hazards model predicting the remating latency. Chi-square values and their *p*-values were calculated with likelihood ratio tests.

Predictors	HR	SE	*p*-value	X^2^	*p*(X^2^)
Vortexing	1.02	0.19	0.94	0.01	0.93
Food	1.14	0.18	0.48	0.49	0.48
No of rivals	1.07	0.18	0.71	0.14	0.71
Replicate	1.09	0.19	0.64	0.22	0.64
Recovery period	1.00	0.01	0.91	0.01	0.91

## Discussion

We tested whether *D. melanogaster* males adjust their reproductive strategies in response to short-term (1 hr) changes in the number of rivals, food availability, and/or experience of mechanical stress before mating. Females mated to males kept in groups of 30 produced more offspring compared to females mated to males kept in groups of 10. Males also varied their copulation duration in response to the number of rivals, but in a condition-dependent manner: in the absence of vortexing, males mated for a shorter time when previously kept with 30 rivals, but the opposite was observed when males were vortexed. We also found that mating latency was affected in a condition-dependent manner in response to competition levels; when males were fed and previously held in groups of 30, they had an increased mating latency compared to the other treatments. These complex responses highlight the importance of using a multiple-stressor approach, as these interactions would not have been seen in a single-stressor experiment.

### Offspring number

Females mated to males housed in groups of 30 produced more offspring than females mated to males housed in groups of 10. This is likely because males vary the amount of sperm and/or SFPs they transfer during mating in response to the number of rivals (Wigby et al. 2009; Garbaczewska et al. 2013; Moatt et al. 2014; Hopkins et al. 2019b). Previous work in *D. melanogaster* has shown that males transfer most sperm when in competition with a single rival, but the abundance of SFPs that are transferred increases with competition levels (Hopkins et al. 2019b). However, it is worth noting that this relationship was only tested with up to eight rivals, which is less than our low-competition treatment. Seminal fluid proteins, particularly sex peptide and ovulin, are responsible for many changes in female behavior after mating, including stimulating and increasing ovulation and egg laying (Wigby et al. 2009; Bretman et al. 2013).

However, there are two alternative explanations for the effect of male rival number on offspring production. First, there is the possibility that males from the two competition treatments smell different to females. Female fruit flies assess male attractiveness partly through their cuticular hydrocarbon (CHC) profile (Grillet et al. 2006; Doubovetzky et al. 2023; Peckenpaugh 2023), which can be transferred between males through physical contact (Coyne et al. 1994; Friberg 2006). Hence, females may perceive differences in the males CHC profiles and exert cryptic female choice by differentially laying eggs or using sperm based on this information. If this was the case, we would expect the effect of male rival number treatment on offspring production to be stronger when males were vortexed, as vortexing increases the transfer of CHCs between males (Kuo et al. 2012; Pischedda et al. 2014). However, we found no interactive effect of the number of rivals and vortexing on offspring number, which suggests that the impacts of rival number on offspring production are unlikely to driven by differential CHC mixes on males.

Second, the familiarity of the males was different between the two rival number treatments. Males in groups of 10 were in an environment with the same males they had been maintained with from eclosion. Males in groups of 30 had 20 novel males introduced to their environment for the hour prior to mating. Unfamiliarity prior to mating could have caused the results found here in response to rival number treatments. However, it has been shown in *D. melanogaster* that rival relatedness is required in addition to familiarity, to modulate male impacts on female reproduction (Le Page et al. 2017).

Our results demonstrate that males can respond much more rapidly to changes in the social environment than previously thought. For example, previous studies in *D. melanogaster* found that more than 24 h of exposure to rivals is needed to see a change in male reproductive behavior (Bretman et al. 2010; Moatt et al. 2014; Rouse and Bretman 2016). This contrasts with our experiment, in which males were held with rivals for only 1 h prior to mating. Even more rapid adjustment of reproductive investments in response to the social environment has been demonstrated in other insect species, such as the beetle *Tenebrio molitor* (Gage and Baker 1991). In this species, male beetles that were subjected to both long-term (5 d) and short-term (5 min) exposure to intrasexual competitors adjusted the number of sperm they inseminated (Gage and Baker 1991). Our findings may differ from previous studies because of the large number of rivals we used: 30 in the high competition treatment and 10 in the low treatment. This contrasts with most previous studies which typically compare isolated males with males held in groups of 2, 4, or 8 (Bretman et al. 2009; Wigby et al. 2009; Bretman et al. 2013a, 2013b; Rouse and Bretman 2016; Hopkins et al. 2019b). Hence our “low” treatment consisted of a higher number of rivals than the “high” treatment of many previous studies. Males may be integrating the number of rivals, the exposure time to them and the time from exposure, in order to assess the magnitude of competition and form an optimal response (Bretman et al. 2023). There is evidence in other species that males may “sum” the total number of rivals encountered over time (Carazo et al. 2012; Lymbery et al. 2019). This suggests that plastic responses may be rapid only when environmental stimuli are extreme enough to warrant such a quick reaction.

### Mating latency

We found suggestive evidence of an interaction between food availability and rival number on the latency to mate: when males were fed and held in groups of 30, they had an increased mating latency compared to the other treatments, although it was marginally non-significant. This provides tentative evidence that males are expressing condition-dependent reproductive behaviors in response to competition. When males are in high quality environments, they may be less willing to invest in matings where the intensity of sperm competition is high (Parker et al. 1996, 1997; Pilastro et al. 2002; Engqvist and Reinhold 2005; Simmons et al. 2007; Thomas and Simmons 2009). However, when males are deprived of food and water, it may signal a reduced likelihood of survival if conditions persist (Good and Tatar 2001). Therefore, it may be advantageous to invest in immediate reproductive effort, despite high levels of competition.

### Copulation duration

Copulation duration depended on an interaction between rival number and vortexing. When males were not vortexed before mating, males that were held in groups of 30 had shorter matings than those held in groups of 10. Although males are likely increasing their investment in SFPs in response to increasing competition, the opposite is true for copulation duration. Copulation duration could be representative of sperm transfer, as a decrease in copulation duration with increasing competition levels fits with early sperm competition theory and empirical research, which predicts that males should reduce their sperm transfer when they directly compete with many rivals (Parker et al. 1996, 1997; Pilastro et al. 2002; Engqvist and Reinhold 2005; Simmons et al. 2007; Thomas and Simmons 2009; Hopkins et al. 2019b). It is not unusual to see differential investment in SFPs and sperm in response to competition levels, as the ejaculate is not a homogeneous entity, and males have independent control over sperm and SFPs (Hopkins et al. 2019b). However, when males were vortexed, males that were held in groups of 30 had longer matings than those held in groups of 10. This suggests that being in physical danger changes the ability of males to respond optimally to changes in the social environment.

## Conclusions

In conclusion, we show that using a multiple stressor approach has many merits in ecological research, particularly given the rapid environmental change natural populations are facing. First, multiple stressor approaches highlight the complexity of the relationship between environment and behavior. In particular, multiple stressor experiments can demonstrate condition-dependent behaviors which helps to reveal the fitness costs associated with these behaviors. Secondly, adopting a multiple stressor approach is beneficial as it more realistically replicates the stress that animals face in nature, with complex fluctuations in stressors. Our study also gave males little time to recover between stressors, and so reveals that reproductive plasticity can occur more rapidly than previously demonstrated.

## Supplementary Material

araf032_suppl_Supplementary_Figure_S1

araf032_suppl_Supplementary_Figure_S2

araf032_suppl_Supplementary_Tables_S1-S10

## Data Availability

Analyses reported in this article can be reproduced using the data provided by Amos et al (2025).
