# Supplementary figures and images for: Short-term increases in rival number improves single mating productivity in male Drosophila
Source: Behav Ecol. 2025 Apr 10;36(3):araf032. doi: 10.1093/beheco/araf032 (PMC12080552; doi:10.1093/beheco/araf032)

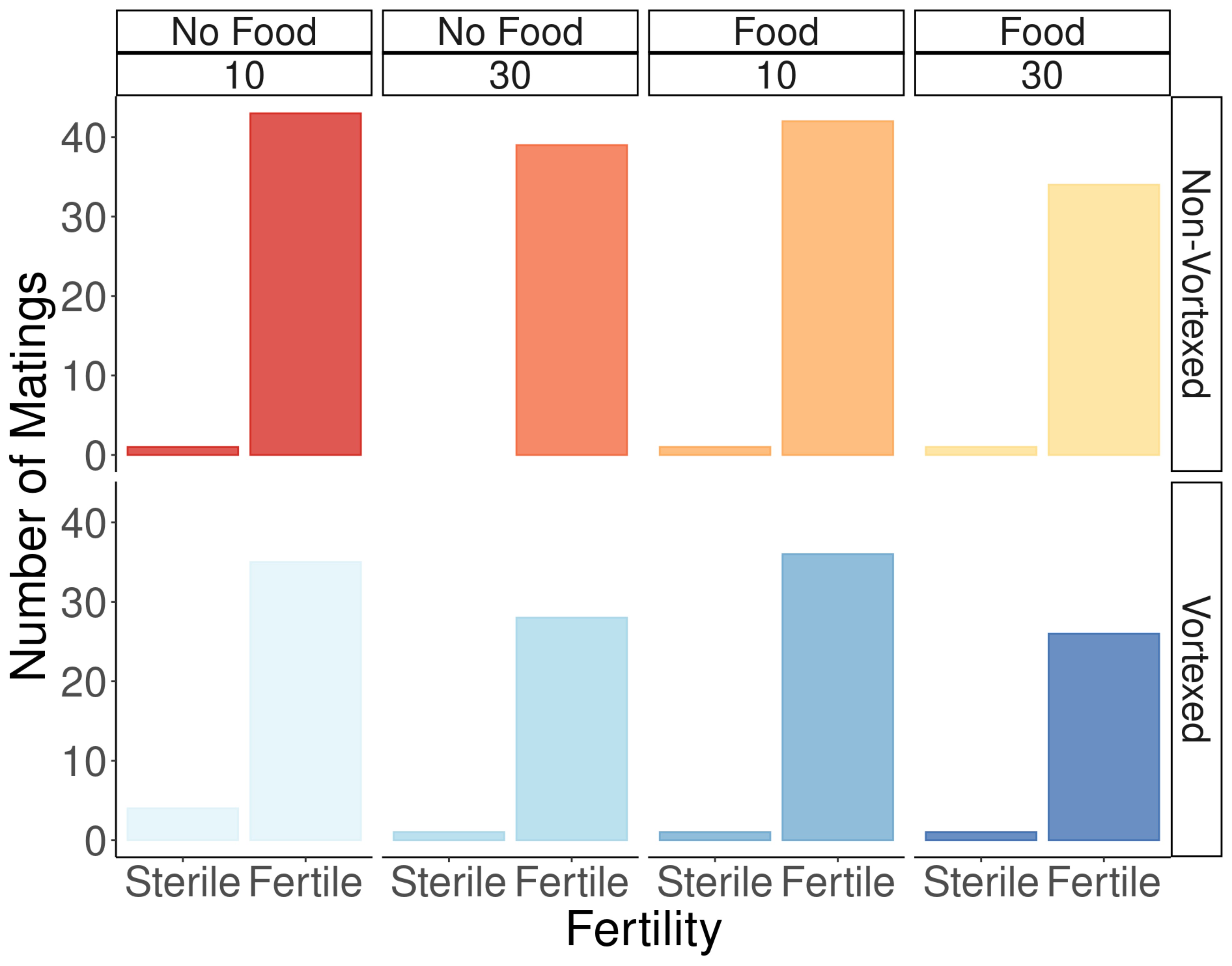

Supplement: araf032_suppl_Supplementary_Figure_S1 [file araf032_suppl_supplementary_figure_s1.jpeg]

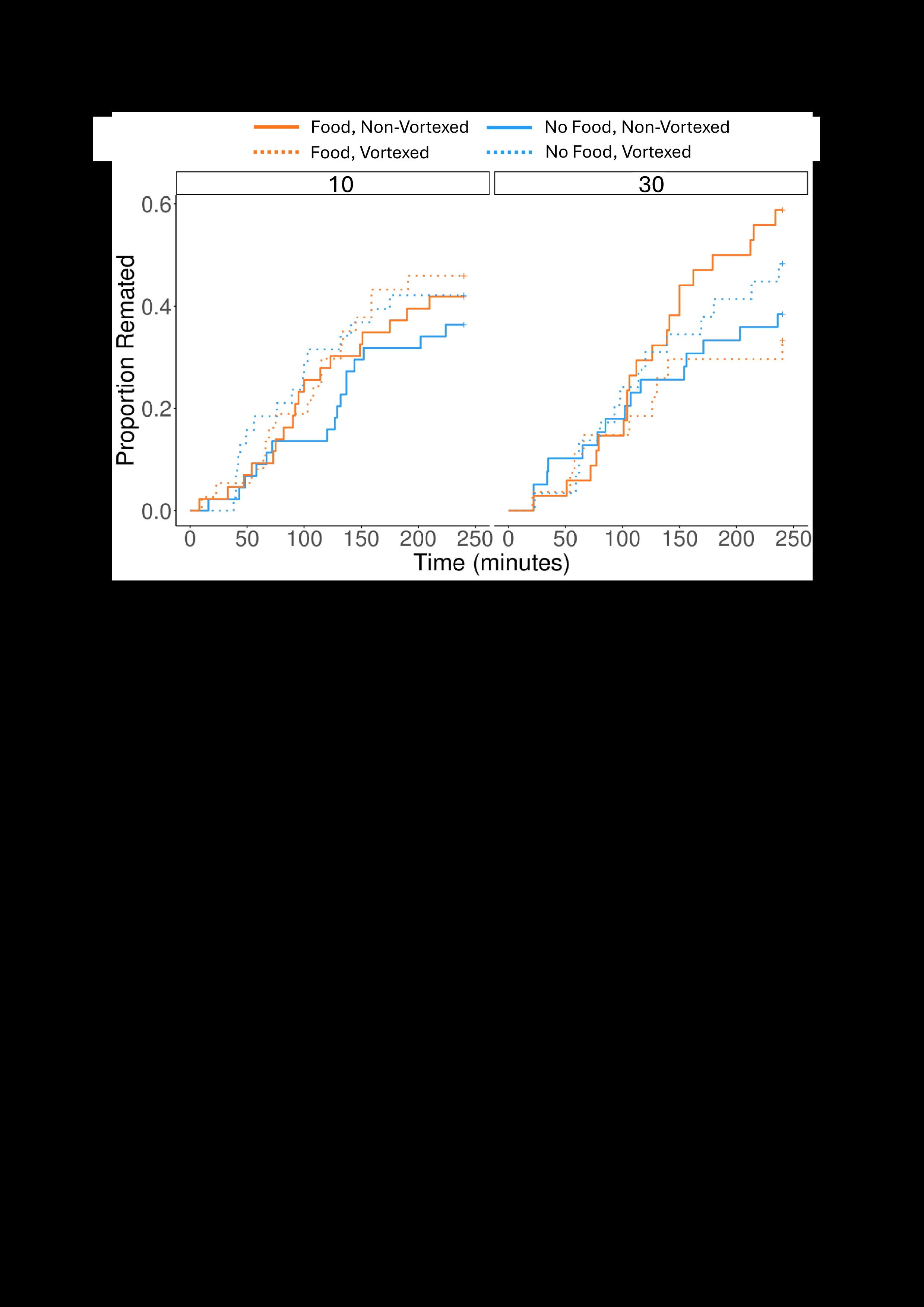

Supplement: araf032_suppl_Supplementary_Figure_S2 [file araf032_suppl_supplementary_figure_s2.jpeg]
