# Supplementary material for: Short-term increases in rival number improves single mating productivity in male Drosophila
Source: Behav Ecol. 2025 Apr 10;36(3):araf032. doi: 10.1093/beheco/araf032 (PMC12080552; doi:10.1093/beheco/araf032)
Supplement: araf032_suppl_Supplementary_Tables_S1-S10 [file araf032_suppl_supplementary_tables_s1-s10.docx]

**Supplementary Material**

**Table S1:** Summary model output for the effect of vortexing, food, number of rivals, replicate, time since the start of the recovery period, and all two-way and three-way interactions between vortexing, food and number of rivals on the mating latency of virgin males. Standard errors and *p-*values were obtained from the model summary, while chi-square values and their *p*-values were calculated with likelihood ratio tests. Significant results are in bold.

| **Predictors** | **HR** | **SE** | ***p*** | ***X^2^*** | ***p(X^2^)*** |
| --- | --- | --- | --- | --- | --- |
| Vortexing | 1.93 | 1.20 | 0.58 | 0.30 | 0.58 |
| Food | 5.10 | 1.22 | 0.18 | 1.79 | 0.18 |
| No of rivals | 1.85 | 1.17 | 0.60 | 0.28 | 0.60 |
| Replicate | 0.85 | 0.12 | 0.18 | 1.78 | 0.18 |
| Recovery period | 1.00 | 0.002 | 0.72 | 0.13 | 0.72 |
| Vortexing * Food | 0.45 | 0.81 | 0.32 | 0.98 | 0.32 |
| Vortexing * No of rivals | 0.87 | 0.76 | 0.85 | 0.04 | 0.85 |
| Food * No of rivals | 0.41 | 0.77 | 0.25 | 1.34 | 0.25 |
| Vortexing * Food * No of rivals | 1.35 | 0.51 | 0.56 | 0.34 | 0.56 |

**Table S2:** Summary model output for the effect of vortexing, food, number of rivals, replicate, time since the start of the recovery period, and all two-way interactions between vortexing, food and number of rivals on the mating latency of virgin males. Standard errors and *p-*values were obtained from the model summary, while chi-square values and their *p*-values were calculated with likelihood ratio tests. Significant results are in bold.

| **Predictors** | **HR** | **SE** | ***p*** | ***X^2^*** | ***p(X^2^)*** |
| --- | --- | --- | --- | --- | --- |
| Vortexing | 1.02 | 0.50 | 0.97 | 0.002 | 0.97 |
| Food | 2.66 | 0.50 | **0.05** | 3.76 | **0.05** |
| No of rivals | 1.00 | 0.52 | 0.99 | 0.00 | 0.99 |
| Replicate | 0.85 | 0.12 | 0.18 | 1.83 | 0.18 |
| Recovery period | 1.00 | 0.002 | 0.90 | 0.02 | 0.90 |
| Vortexing * Food | 0.71 | 0.24 | 0.15 | 2.04 | 0.15 |
| Vortexing * No of rivals | 1.32 | 0.26 | 0.28 | 1.16 | 0.28 |
| Food * No of rivals | 0.63 | 0.23 | **0.05** | 3.96 | **0.05** |

**Table S3:** Summary model output for the effect of vortexing, food, number of rivals, replicate, time since the start of the recovery period and all two-way and three-way interactions between vortexing, food and number of rivals on the copulation duration. Standard errors and *p-*values were obtained from the model summary, while *F-*values and their *p*-values were calculated with likelihood ratio tests. Significant results are in bold.

| **Predictors** | **Estimate** | **SE** | ***p*** | ***F*** | ***p(F)*** |
| --- | --- | --- | --- | --- | --- |
| Intercept | 20.78 | 1.86 |  |  |  |
| Vortexing (Vortexed) | - 2.20 | 0.84 | **0.01** | 6.80 | **0.01** |
| Food (Food) | - 1.09 | 0.82 | 0.19 | 1.77 | 0.19 |
| No of rivals (30) | - 2.13 | 0.83 | **0.01** | 6.62 | **0.01** |
| Replicate (2) | - 0.78 | 0.44 | 0.08 | 3.14 | 0.08 |
| Recovery period | -0.00 | 0.01 | 0.78 | 0.08 | 0.78 |
| Vortexing * Food | 1.74 | 1.33 | 0.19 | 1.71 | 0.19 |
| Vortexing (Vortexed) * No of rivals (30) | 3.35 | 1.24 | **0.01** | 7.35 | **0.01** |
| Food * No of rivals | 1.35 | 1.21 | 0.26 | 1.25 | 0.26 |
| Vortexing * Food * No of rivals | - 2.06 | 1.90 | 0.28 | 1.18 | 0.28 |

**Table S4:** Summary model output for the effect of vortexing, food, number of rivals, replicate, time since the start of the recovery period and all two-way interactions between vortexing, food and number of rivals on the copulation duration. Standard errors and *p-*values were obtained from the model summary, while *F-*values and their *p*-values were calculated with likelihood ratio tests. Significant results are in bold.

| **Predictors** | **Estimate** | **SE** | ***p*** | ***F*** | ***p(F)*** |
| --- | --- | --- | --- | --- | --- |
| Intercept | 21.30 | 1.79 |  |  |  |
| Vortexing (Vortexed) | - 1.74 | 0.73 | **0.02** | 5.68 | **0.02** |
| Food (Food) | - 0.65 | 0.72 | 0.36 | 0.83 | 0.36 |
| No of rivals (30) | - 1.75 | 0.75 | **0.02** | 5.44 | **0.02** |
| Replicate (2) | - 0.79 | 0.44 | 0.07 | 3.21 | 0.07 |
| Recovery period | -0.01 | 0.01 | 0.46 | 0.55 | 0.46 |
| Vortexing * Food | 0.70 | 0.92 | 0.45 | 0.58 | 0.45 |
| Vortexing (Vortexed) * No of rivals (30) | 2.54 | 0.98 | **0.01** | 6.67 | **0.01** |
| Food * No of rivals | 0.45 | 0.88 | 0.61 | 0.26 | 0.61 |

**Table S5:** Summary model output for the effect of male vortexing, food, number of rivals, replicate, time since the start of the recovery period and all two-way and three-way interactions between vortexing, food and number of rivals on the proportion of matings that produced any offspring. Standard errors and *p-*values were obtained from the model summary, while chi-square values and their *p*-values were calculated with likelihood ratio tests. Significant results are in bold.

| **Predictors** | **Estimate** | **SE** | ***p*** | **X^2^** | ***p*(X^2^)** |
| --- | --- | --- | --- | --- | --- |
| Intercept | 4.27 | 1.80 |  |  |  |
| Vortexing (Vortexed) | - 1.41 | 1.19 | 0.23 | 1.41 | 0.23 |
| Food (Food) | 0.10 | 1.46 | 0.94 | 0.01 | 0.94 |
| No of rivals (30) | 0.16 | 0.0017 | 0.99 | 0.0001 | 0.99 |
| Replicate (2) | 0.38 | 0.68 | 0.57 | 0.32 | 0.57 |
| Recovery period | - 0.0067 | 0.013 | 0.61 | 0.26 | 0.61 |
| Vortexing * Food | 0.92 | 2.13 | 0.67 | 0.19 | 0.67 |
| Vortexing * No of rivals | - 0.15 | 0.0017 | 0.99 | 0.0001 | 0.99 |
| Food * No of rivals | - 0.16 | 0.0017 | 0.99 | 0.0001 | 0.99 |
| Vortexing * Food * No of rivals | 0.15 | 0.0017 | 0.99 | 0.0001 | 0.99 |

**Table S6:** Summary model output for the effect of male vortexing, food, number of rivals, replicate, time since the start of the recovery period and all two-way interactions between vortexing, food and number of rivals on the proportion of matings that produced any offspring. Standard errors and *p-*values were obtained from the model summary, while chi-square values and their *p*-values were calculated with likelihood ratio tests. Significant results are in bold.

| **Predictors** | **Estimate** | **SE** | ***p*** | **X^2^** | ***p*(X^2^)** |
| --- | --- | --- | --- | --- | --- |
| Intercept | 4.15 | 1.78 |  |  |  |
| Vortexing (Vortexed) | - 1.68 | 1.16 | 0.15 | 2.11 | 0.15 |
| Food (Food) | - 0.26 | 1.33 | 0.85 | 0.04 | 0.85 |
| No of rivals (30) | 1.68 | 1.93 | 0.38 | 0.76 | 0.38 |
| Replicate (2) | 0.39 | 0.68 | 0.56 | 0.33 | 0.56 |
| Recovery period | - 0.004 | 0.01 | 0.74 | 0.11 | 0.74 |
| Vortexing * Food | 1.65 | 1.75 | 0.35 | 0.89 | 0.35 |
| Vortexing * No of rivals | - 0.30 | 1.96 | 0.88 | 0.02 | 0.88 |
| Food * No of rivals | - 1.69 | 1.69 | 0.32 | 1.00 | 0.32 |

**Table S7:** Summary model output for the effects of male vortexing, food, number of rivals, replicate, time since the start of the recovery period and all two-way and three-way interactions between vortexing, food and number of rivals on the number of offspring produced by females. This analysis was only run on the subset of females that produced any offspring. Standard errors and *p-*values were obtained from the model summary, while *F*-values and their *p*-values were calculated with likelihood ratio tests. Significant results are in bold.

| **Predictors** | **Estimate** | **SE** | ***p*** | ***F*** | ***p*(F)** |
| --- | --- | --- | --- | --- | --- |
| Intercept | 55.42 | 3.79 |  |  |  |
| Vortexing (Vortexed) | 2.35 | 3.18 | 0.46 | 0.55 | 0.46 |
| Food (Food) | 3.70 | 3.05 | 0.23 | 1.47 | 0.23 |
| No of rivals (30) | 6.51 | 3.03 | 0.03 | 4.62 | **0.03** |
| Replicate (2) | 3.30 | 1.65 | 0.05 | 4.02 | **0.05** |
| Recovery period | - 0.06 | 0.03 | 0.05 | 4.05 | 0.05 |
| Vortexing * Food | - 2.41 | 5.00 | 0.63 | 0.23 | 0.63 |
| Vortexing * No of rivals | 0.06 | 4.63 | 0.99 | 0.00 | 0.99 |
| Food * No of rivals | - 5.35 | 4.49 | 0.23 | 1.42 | 0.23 |
| Vortexing * Food * No of rivals | 5.66 | 7.15 | 0.43 | 0.63 | 0.43 |

**Table S8:** Summary model output for the effects of male vortexing, food, number of rivals, replicate, time since the start of the recovery period and all two-way interactions between vortexing, food and number of rivals on the number of offspring produced. This analysis was only run on the subset of females that produced any offspring. Standard errors and *p-*values were obtained from the model summary, while *F*-values and their *p*-values were calculated with likelihood ratio tests. Significant results are in bold.

| **Predictors** | **Estimate** | **SE** | ***p*** | ***F*** | ***p*(F)** |
| --- | --- | --- | --- | --- | --- |
| Intercept | 54.99 | 3.75 |  |  |  |
| Vortexing (Vortexed) | 1.10 | 2.75 | 0.69 | 0.16 | 0.69 |
| Food (Food) | 2.52 | 2.66 | 0.34 | 0.90 | 0.34 |
| No of rivals (30) | 5.52 | 2.76 | **0.05** | 4.01 | **0.05** |
| Replicate (2) | 3.31 | 1.64 | **0.04** | 4.06 | **0.04** |
| Recovery period | - 0.05 | 0.03 | 0.07 | 3.43 | 0.06 |
| Vortexing * Food | 0.44 | 3.45 | 0.90 | 0.02 | 0.90 |
| Vortexing * No of rivals | 2.27 | 3.69 | 0.54 | 0.38 | 0.54 |
| Food * No of rivals | - 2.93 | 3.28 | 0.37 | 0.80 | 0.37 |

**Table S9:** Summary model output for the effect of first-male vortexing, food, number of rivals, replicate, time since the start of the recovery period and all two-way and three-way interactions between vortexing, food and number of rivals on the remating latency. Standard errors and *p-*values were obtained from the model summary, while chi-square values and their *p*-values were calculated with likelihood ratio tests. Significant results are in bold.

| **Predictors** | **HR** | **SE** | ***p*** | ***X^2^*** | ***p(X^2^)*** |
| --- | --- | --- | --- | --- | --- |
| Vortexing | 0.51 | 1.85 | 0.72 | 0.13 | 0.72 |
| Food | 0.35 | 1.81 | 0.56 | 0.34 | 0.56 |
| No of rivals | 0.30 | 1.84 | 0.51 | 0.44 | 0.51 |
| Replicate | 1.03 | 0.20 | 0.89 | 0.02 | 0.89 |
| Recovery period | 1.00 | 0.01 | 0.53 | 0.40 | 0.53 |
| Vortexing * Food | 2.52 | 1.21 | 0.44 | 0.59 | 0.44 |
| Vortexing * No of rivals | 2.74 | 1.22 | 0.41 | 0.68 | 0.41 |
| Food * No of rivals | 3.71 | 1.81 | 0.27 | 1.23 | 0.27 |
| Vortexing * Food * No of rivals | 0.38 | 0.80 | 0.22 | 1.49 | 0.22 |

**Table S10:** Summary model output for the effect of first-male vortexing, food, number of rivals, replicate, time since the start of the recovery period and all two-way interactions between vortexing, food and number of rivals on the remating latency. Standard errors and *p-*values were obtained from the model summary, while chi-square values and their *p*-values were calculated with likelihood ratio tests. Significant results are in bold.

| **Predictors** | **HR** | **SE** | ***p*** | ***X^2^*** | ***p(X^2^)*** |
| --- | --- | --- | --- | --- | --- |
| Vortexing | 3.92 | 0.82 | 0.10 | 2.78 | 0.10 |
| Food | 2.54 | 0.82 | 0.26 | 1.29 | 0.26 |
| No of rivals | 2.17 | 0.85 | 0.36 | 0.83 | 0.36 |
| Replicate | 1.07 | 0.19 | 0.73 | 0.12 | 0.73 |
| Recovery period | 1.00 | 0.01 | 0.86 | 0.03 | 0.86 |
| Vortexing * Food | 0.62 | 0.37 | 0.19 | 1.76 | 0.19 |
| Vortexing * No of rivals | 0.66 | 0.37 | 0.27 | 1.22 | 0.27 |
| Food * No of rivals | 0.94 | 0.37 | 0.87 | 0.03 | 0.87 |
